# Supplementary material for: Phenotypic and functional characterization of tumor-reactive T cells in malignant pleural effusions
Source: bioRxiv. 2025 Dec 9:2025.12.05.692662. Preprint. [Version 1] doi: 10.64898/2025.12.05.692662 (PMC12709480; doi:10.64898/2025.12.05.692662)
Supplement: Supplement 1 [file media-1.pdf]

Figure S1

(A) *PBMCS*

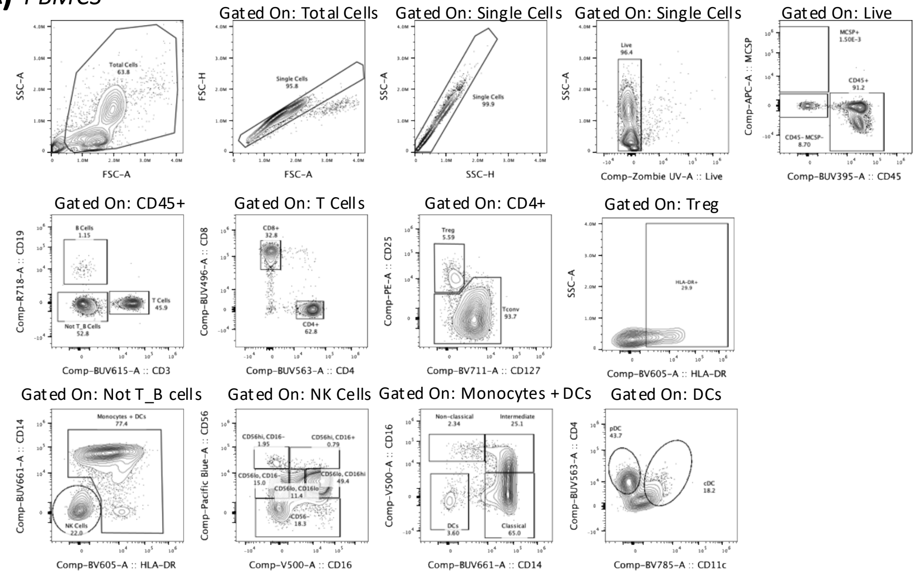

(B) *Ex vivo MPE*

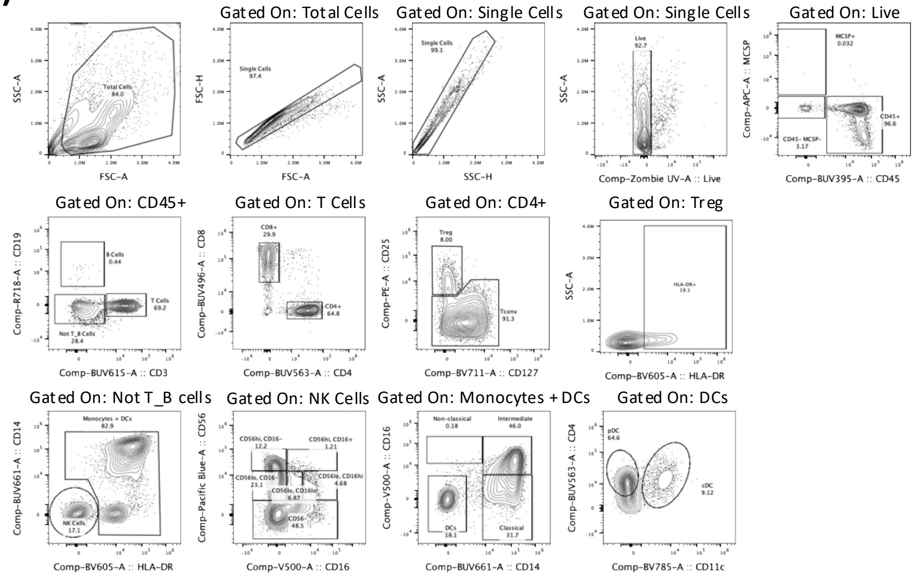

(C) *Tumor*

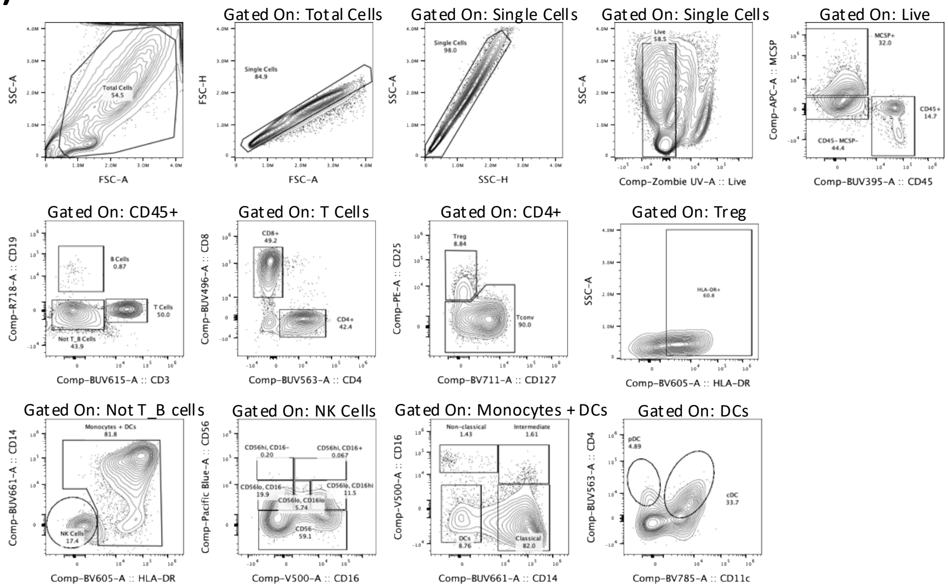

Flow cytometry gating strategy to analyze *ex vivo* immune cell populations in (A) peripheral blood mononuclear cells (PBMC), (B) malignant pleural effusions (MPE), and (C) tumor.

Figure S2

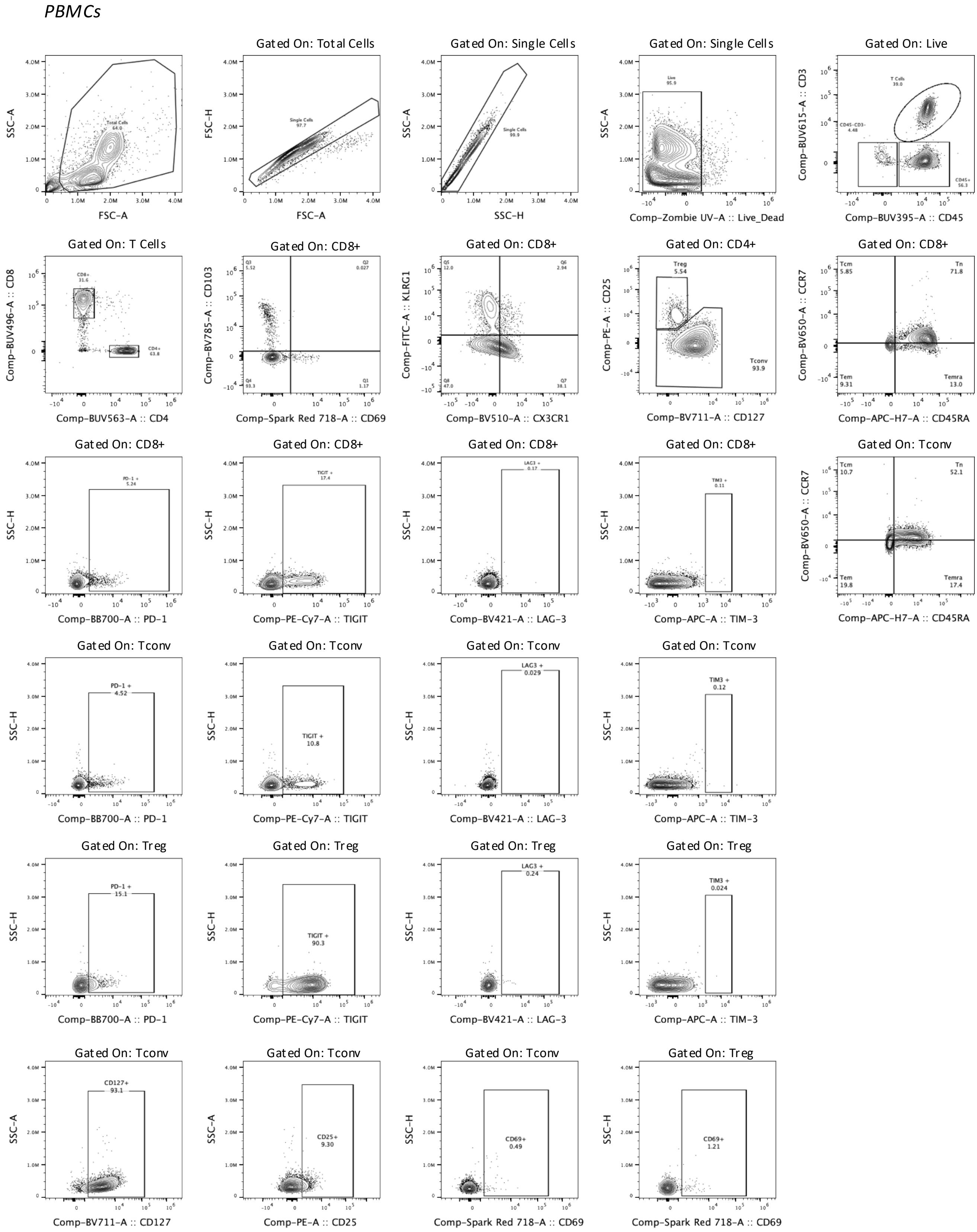

Flow cytometry gating strategy to analyze T cell populations and co-inhibitory receptor expression in PBMC.

Figure S3

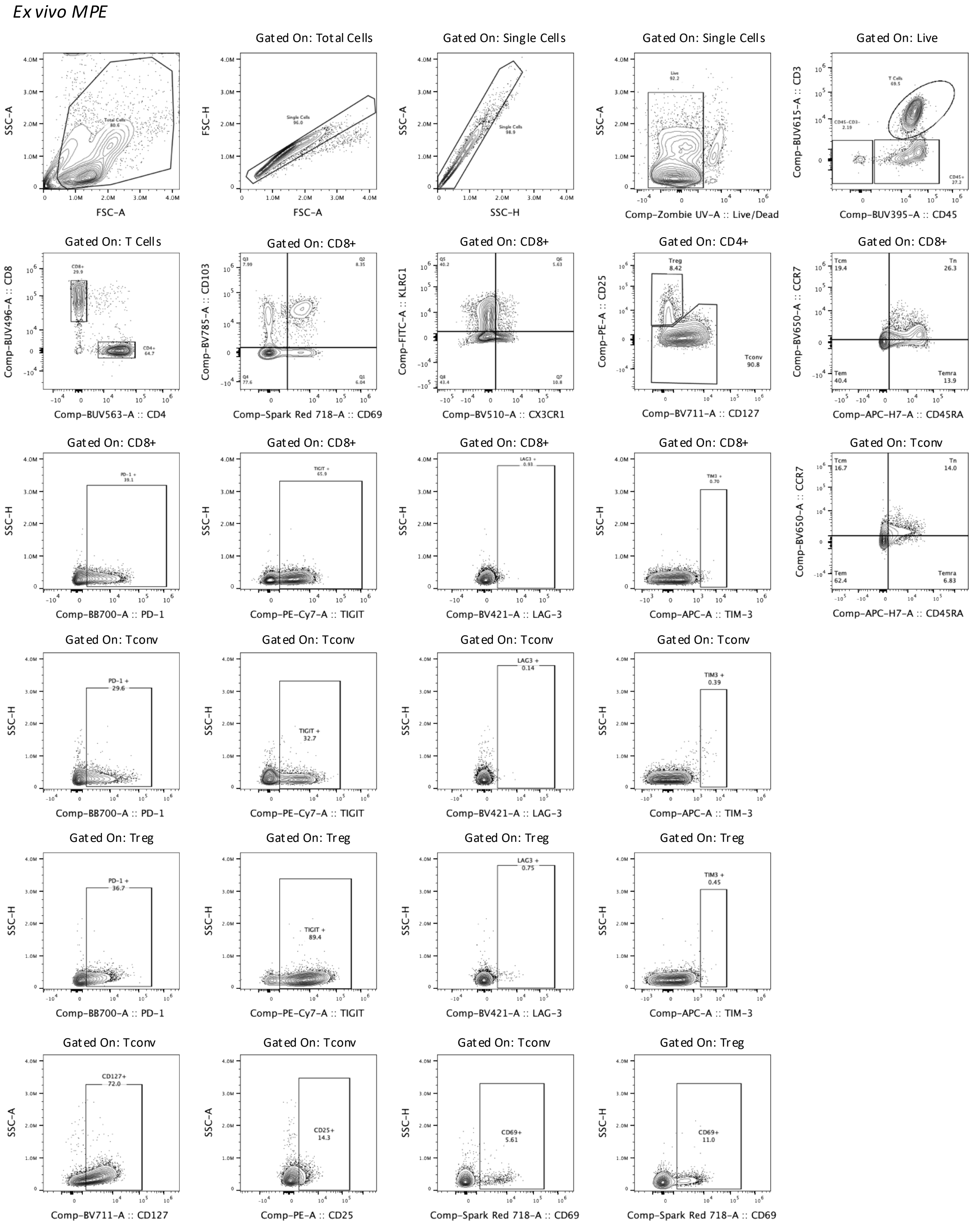

Flow cytometry gating strategy to analyze T cell populations and co-inhibitory receptor expression in *ex vivo* MPE.

Figure S4

Tumor

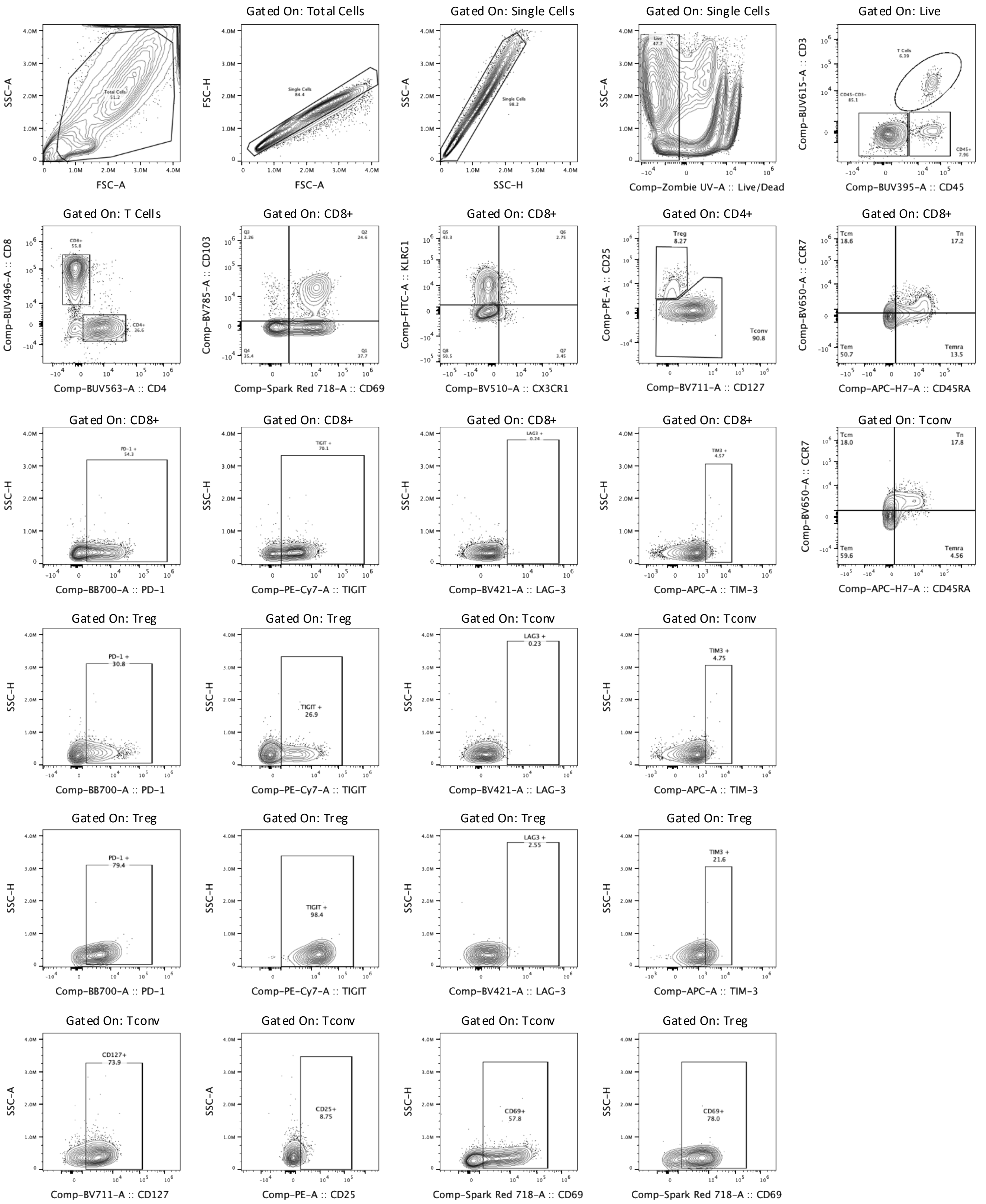

Flow cytometry gating strategy to analyze T cell populations and co-inhibitory receptor expression in tumor.

Figure S5

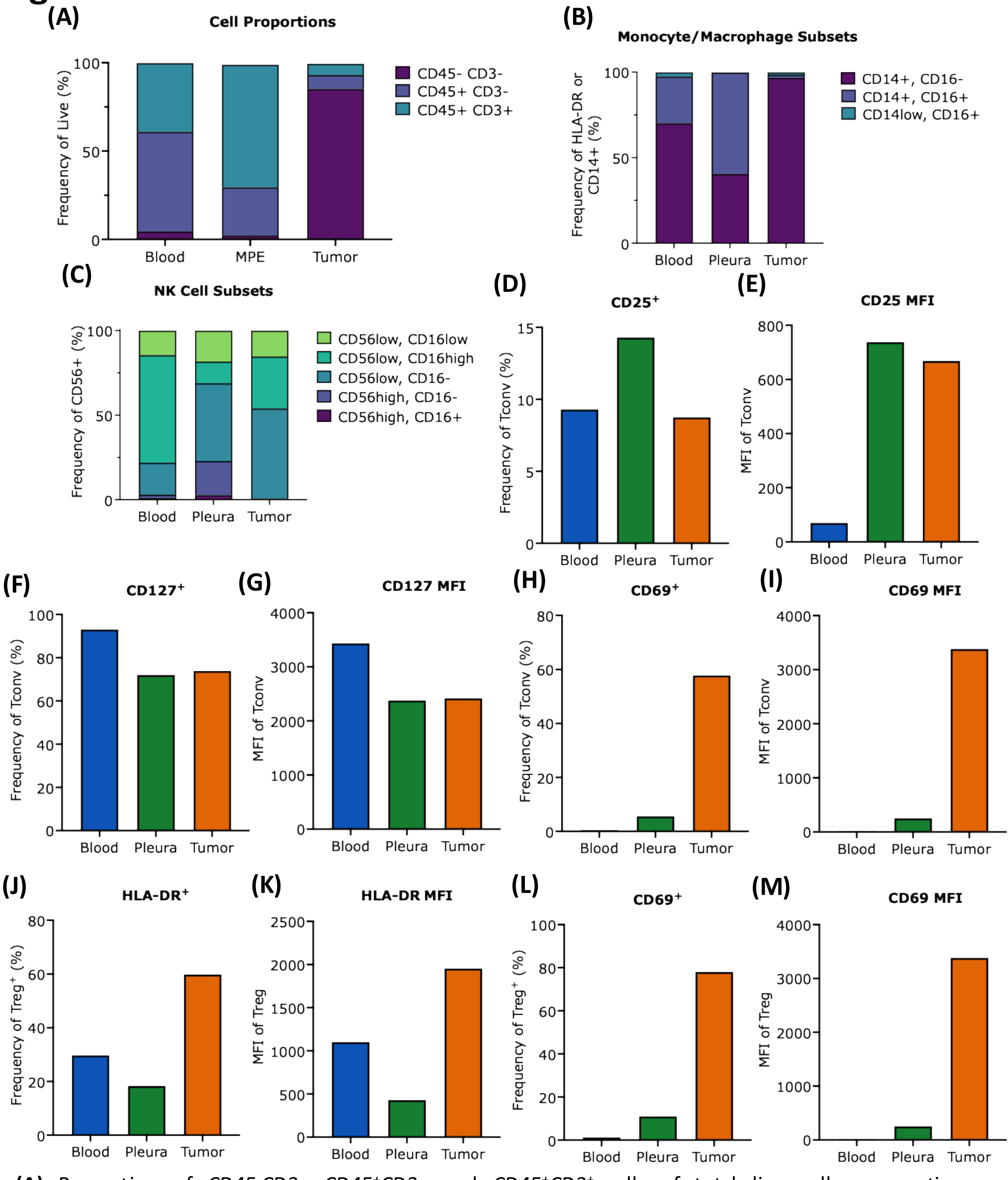

(A) Proportion of *CD45⁻CD3⁻*, *CD45⁺CD3⁻*, and *CD45⁺CD3⁺* cells of total live cells across tissue compartments. **(B)** Proportion of *CD14⁺CD16⁻*, *CD14⁺CD16⁺*, and *CD14^low^CD16⁺* monocytes/macrophages across tissue compartments as a percentage of total monocytes/macrophages. **(C)** Proportion of *CD56^high^CD16⁻*, *CD56^high^CD16⁺*, *CD56^low^CD16⁻*, *CD56^low^CD16^low^*, and *CD56^low^CD16^high^* NK cell populations across tissue compartments as a percentage of *CD56⁺* cells. **(D)** *CD25* expression on conventional *CD4⁺CD25^low/–^*(Tconv) T cells by frequency and **(E)** mean fluorescence intensity (MFI). **(F)** *CD127* expression on Tconv cells by frequency and **(G)** MFI. **(H)** *CD69* expression on Tconv cells by frequency and **(I)** MFI. **(J)** *HLA-DR* expression on regulatory *CD4⁺CD25^high^CD127^low^* (Treg) T cells by frequency and **(K)** MFI. **(L)** *CD69* expression on Treg cells by frequency and (M) MFI.

Figure S6

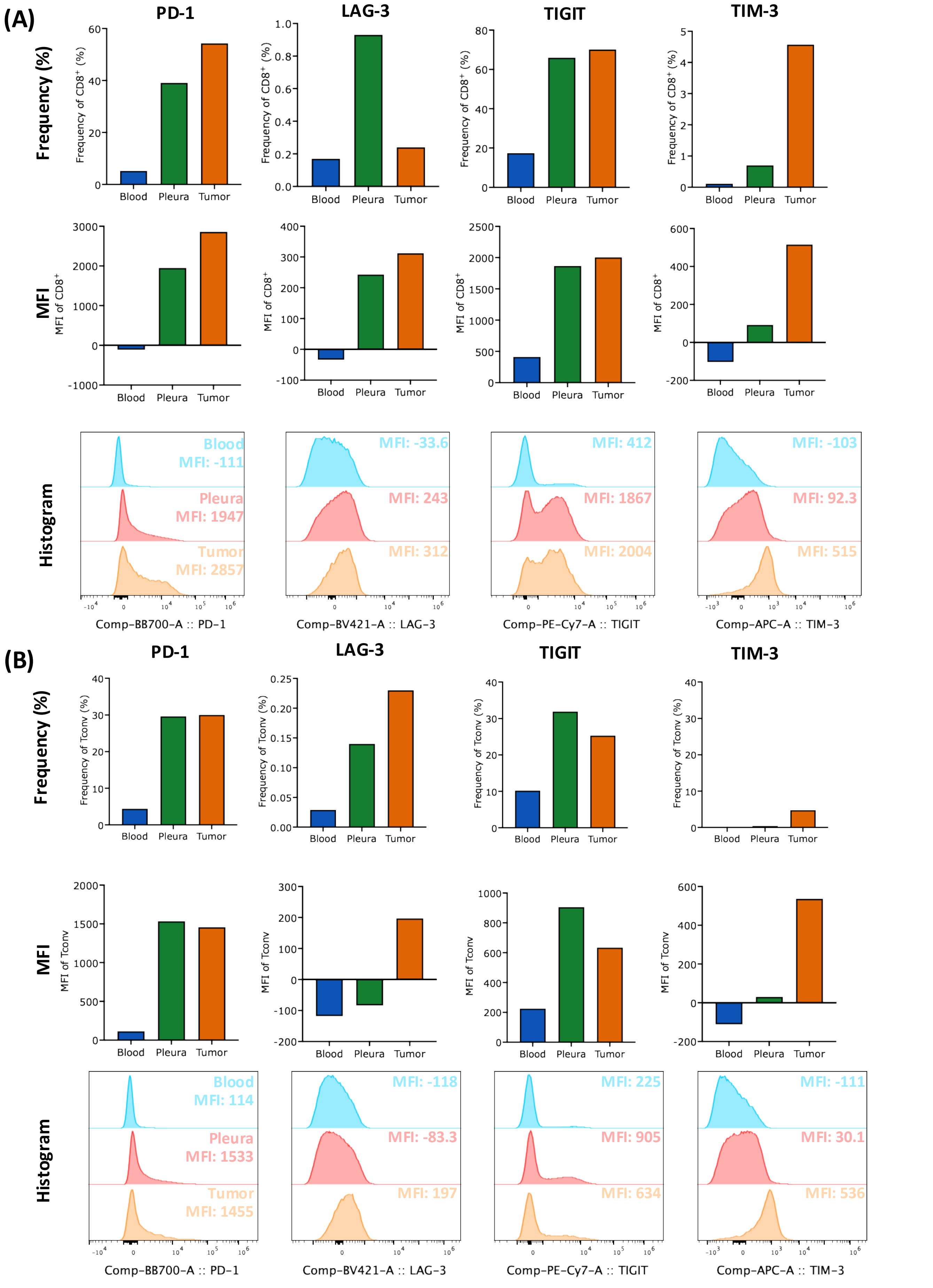

(A) Frequency, mean fluorescence intensity (MFI), and histograms showing expression of *PD-1*, *LAG-3, TIGIT*, and *TIM-3* on *CD8⁺* T cells and **(B)** Conventional *CD4⁺CD25^low/-^* (Tconv) T cells across tissue compartments.

Figure S7

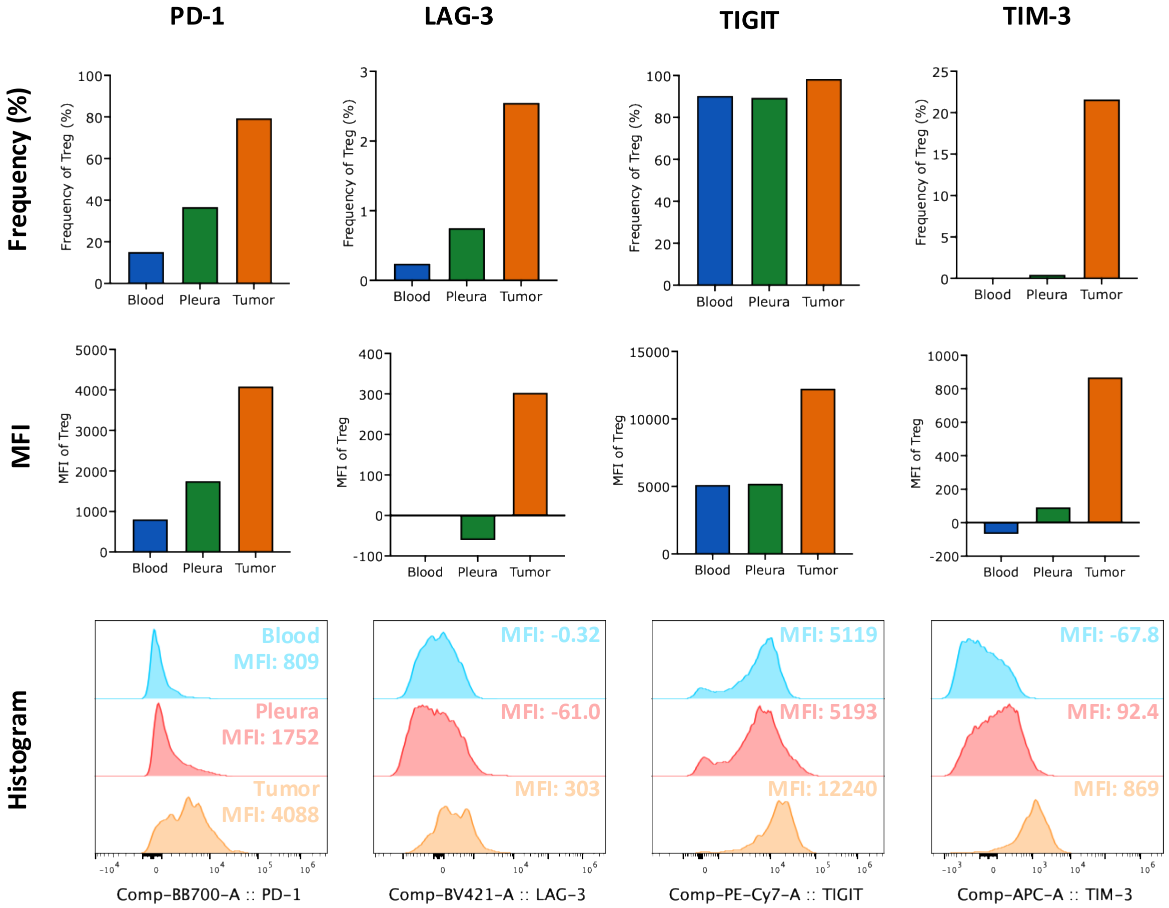

Frequency, mean fluorescence intensity (MFI), and histograms showing expression of *PD-1*, *LAG-3*, *TIGIT*, and *TIM-3* on regulatory $CD4^{+}CD25^{high}CD127^{low}$ (Treg) T cells across tissue compartments

Figure S8

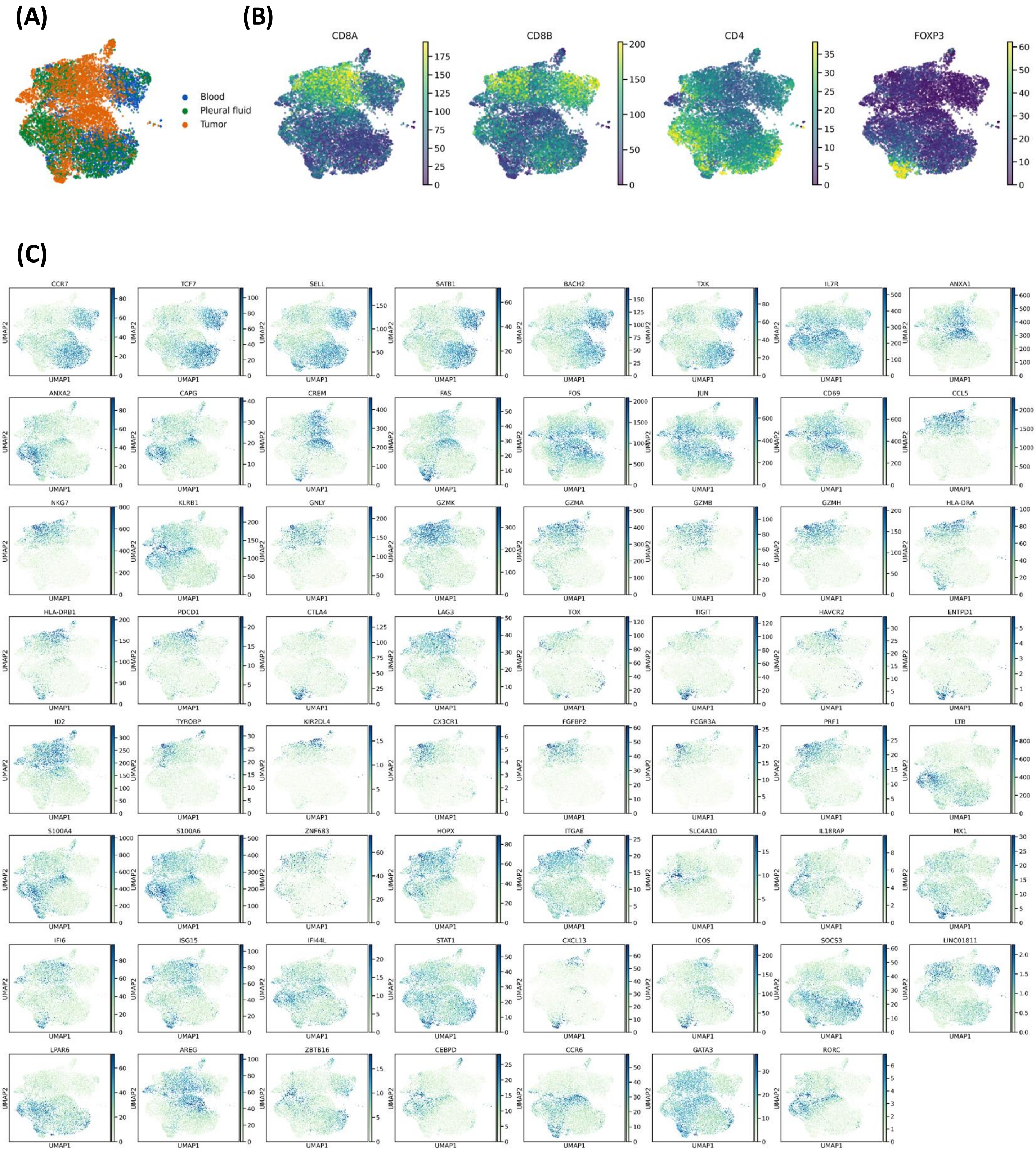

(A) UMAP visualization cells across MPE, blood and tumor **(B)** UMAPs showing expression of canonical T cell markers **(C)** UMAPs showing expression of curated markers

Figure S9

(A) (B)

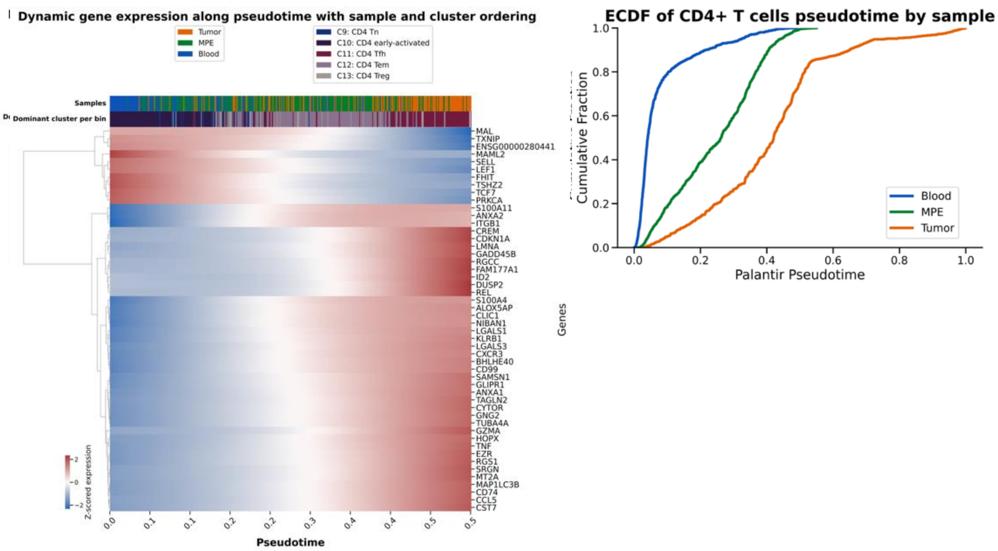

(A) Differential gene expression along pseudotime trajectory of CD4^{+} T cells **(B)** Empirical cumulative distribution function (ECDF) of compartment-specific CD4^{+} T cells along the pseudotime trajectory.

Figure S10

(A) (B)

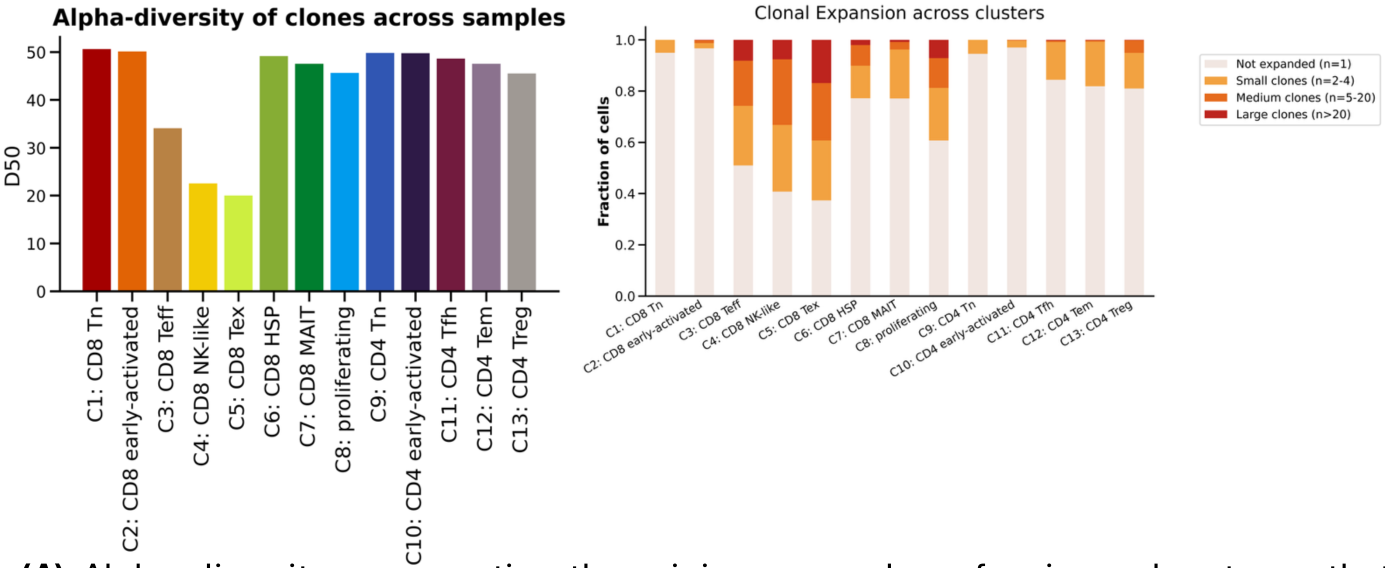

(A) Alpha diversity, representing the minimum number of unique clonotypes that account for 50% of all cells. across clusters. **(B)** Clonal expansion across Clusters.

Figure S11

(A)

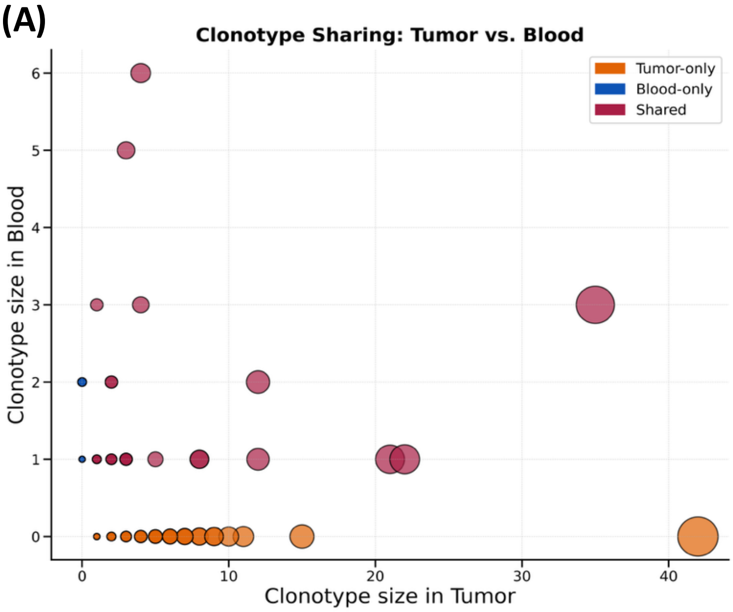

(A) Clonotype sharing between tumor and blood.

Figure S12

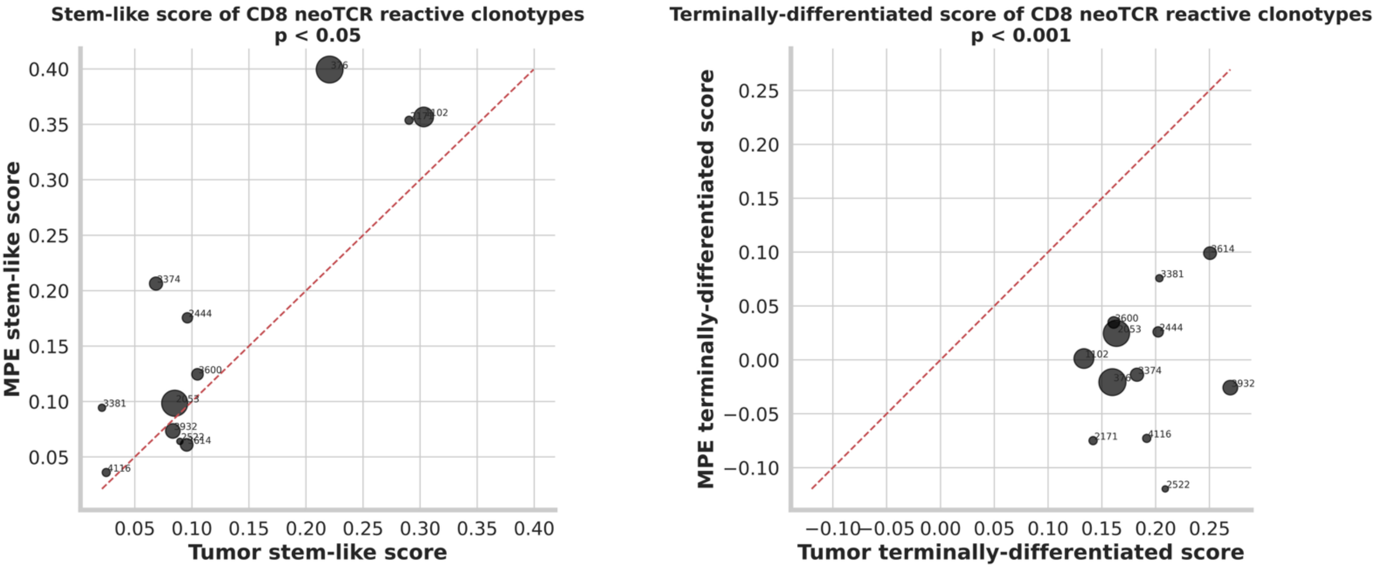

(A) Gene signature scores for stem like and **(B)** terminally differentiated T cells.

Figure S13

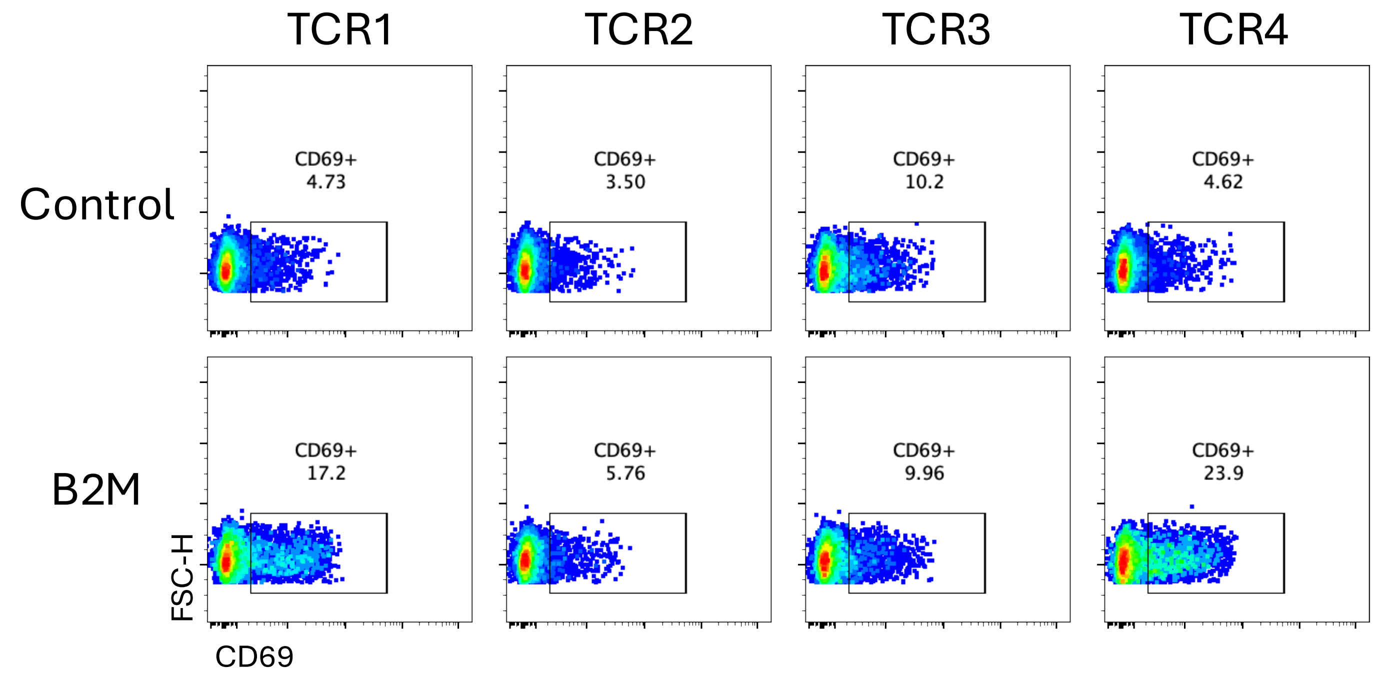

Representative flow cytometry plots of CD69 expression in Jurkat cells when co-cultured with cancer cell lines established from the patient's resected lung metastasis. The Jurkat cells are expressing TCR's identified in clonally expanded T cells identified in both tumor and MPE (TCR1–TCR4). The cell lines were either untransduced (top row) or transduced to re-express *B2M* (bottom row).

Figure S14

(A)

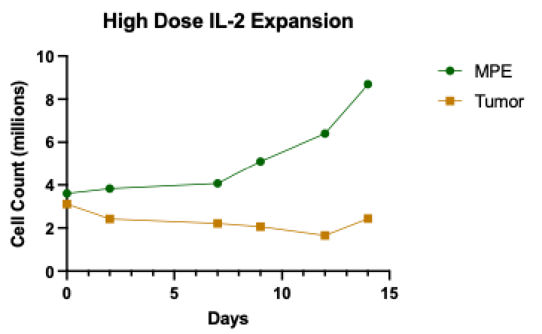

(B)

B2M Pleural Metastasis with Expanded MPE Cells

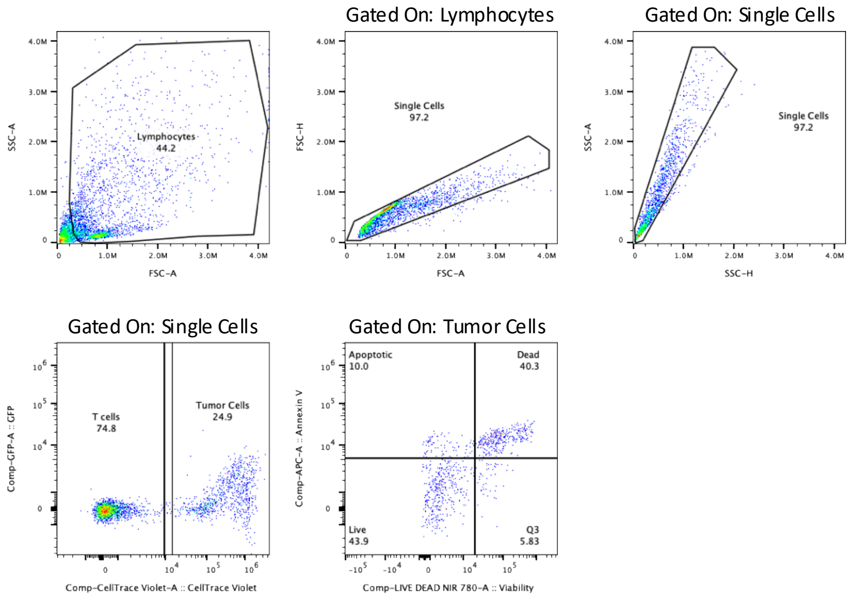

(C)

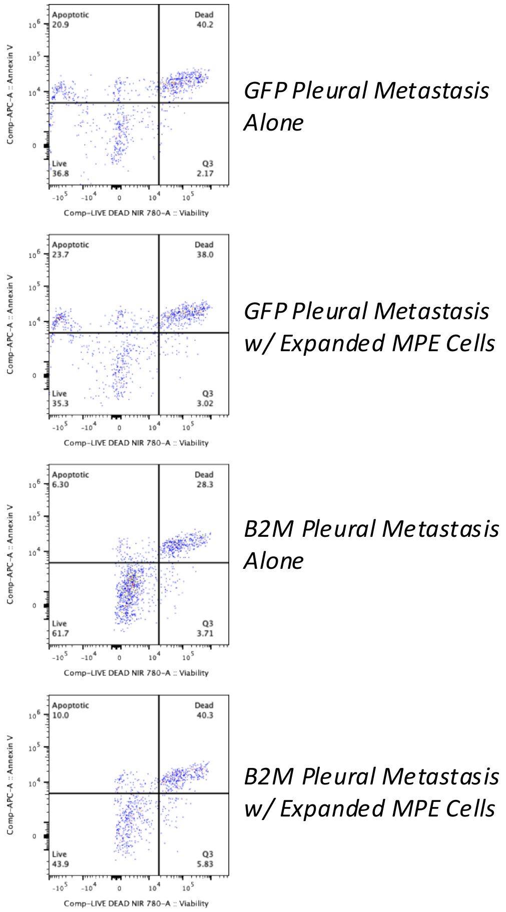

(A) Cell counts of MPE and tumor cells in high-dose IL-2 over the course of 2 weeks.

(B) Gating strategy for determining pleural metastasis cell viability in co-culture assay.

(C) Representative staining patterns of Live/Dead and Annexin V in GFP or B2M transduced pleural metastasis cells alone or with expanded MPE *CD8*$^{+}$ T cells
